# Supplementary material for: Strengthening individual and family resilience against leprosy-related discrimination: A pilot intervention study
Source: PLoS Negl Trop Dis. 2021 Apr 2;15(4):e0009329. doi: 10.1371/journal.pntd.0009329 (PMC8046345; doi:10.1371/journal.pntd.0009329)
Supplement: S1 Text — (DOCX) [file pntd.0009329.s002.docx]

**S1 Supporting information file**

**Table 1. Overview of sessions in the resilience intervention and corresponding dimensions of resilience**

| **Intervention session** | | **Brief description of content of intervention session** | **Alignment with key dimensions** |
| --- | --- | --- | --- |
| 1 | Introduction to resilience | - Introduction to concept of resilience - Insight in what we can control and what we can’t control | - Self-reliance and self-determination (locus of control, problem solving skills) [1] - Problem solving [2] |
| 2 | Knowledge about leprosy | - Demystify misconceptions and reinforce knowledge about leprosy, through a boardgame (adapted ‘snakes and ladders’) - The power of knowledge, knowledge and discrimination | - Accurate information [2,3] |
| 3 | Positive thinking (about self, personal strengths) | - Positive view of yourself and family members, recognize strengths, treating yourself like a friend - Awareness of thoughts and how thoughts can influence emotions and behaviour - Introduction to adapting to change - Positive self-talk | - Sense of purpose and personal efficacy [1] - Self-reliance and self-determination [1] - Positive outlook [4] - Problem solving [2] |
| 4 | Positive thinking (general/situations) | - Recognize how thoughts can affect the ability to cope with situations - Helpful and unhelpful thoughts, our thoughts are not always ‘the truth’ - Identify, regulate and challenge negative and counterproductive thought - Practice being grateful | - Belief systems and values [1] - Locus of control, personal confidence in problem solving skills and abilities [1] - Make meaning of adversity [4] - Positive outlook [4] - Open emotional expression [4] |
| 5 | Accepting changes | - Accepting change - Insight into bodily change as a normal thing in life - Focus on the things the body can physically - Insight in what we can control and what we can’t control | - Personal efficacy [1] - Flexibility [4] - Problem solving [2] |
| 6 | Rights | - The importance of knowledge about rights and accessing rights | - Importance of understanding rights [2] |
| 7 | Beliefs and faith, finding meaning in life | - Focusing on God or a higher power in difficult times can help - Spirituality can be very constructive for dealing with discrimination and exclusion - Some aspects of some religions can also be negative | - Spirituality [1,3] - Transcendence and spirituality [4] |
| 8 | Relationships (inside the family) | - The importance of supporting each other in the family - Create a supportive environment for the whole family | - Family cohesion and warmth [1] - Connectedness [4] - Collaborative problem solving [2] - Family and social relationships [3] |
| 9 | Relationships (outside the family) | - The importance of social relationships for resilience and wellbeing - Things you can do to build social relationships | - Community support [1] - Connectedness [4] - Social and economic resources [4] - Collaborative problem solving [2] - Family and social relationships [3] |
| 10 | Interaction with peers | - Bring people together to discuss the experiences they’ve had with the resilience intervention - Expand friendships and support networks | - Community support [1] - Connectedness [4] - Collaborative problem solving [2] - Peer relationships [3] |

1. Bhana A, Bachoo S. The determinants of family resilience among families in low-and middle-income contexts: a systematic literature review. South African J Psychol 2011;41:131–9.

2. Van’t Noordende AT, Kuipers P, Pereira ZBD. Strengthening personal and family resilience: a literature review for the leprosy context. Lepr Rev 2019;90:88–104.

3. Van’t Noordende AT, Pereira Z, Kuipers P. Sources of strength and resilience for persons affected by leprosy in Brazil: What can we learn for service development? Submitt Publ 2020.

4. Walsh F. Family resilience: A framework for clinical practice. Fam Process 2003;42:1–18.
