# Supplementary material for: Strengthening individual and family resilience against leprosy-related discrimination: A pilot intervention study
Source: PLoS Negl Trop Dis. 2021 Apr 2;15(4):e0009329. doi: 10.1371/journal.pntd.0009329 (PMC8046345; doi:10.1371/journal.pntd.0009329)
Supplement: S2 Text — (DOCX) [file pntd.0009329.s003.docx]

**S2 Supporting information file**

**Median difference per question pre- and post-intervention on the resilience scale (CD-RISC)**

*Each question could be scored as 0 = not true at all, 1 = rarely true, 2 = sometimes true, 3 = often true, 4 = true nearly all the time.*


**Median difference per question pre- and post-intervention on** **the quality of life scale (WHOQOL-BREF)**

*Each question could be scored as = 1 very dissatisfied/not at all, 5 = very satisfied/completely. Last question (Q26): 1=never, 5=always.*

**Difference** **in raw scores per domain on the WHOQOL-BREF, pre- and post-intervention**

**Odisha state**

|  | **Median and IQR baseline score (n=39)** | **Median and IQR follow-up sore (n=39)** | **Difference baseline vs follow-up (%)** | **p-value^a^** |
| --- | --- | --- | --- | --- |
| Physical health (range 7-35) | 21.0 (20.0-23.0) | 26.0 (25.0-28.0) | 5.0 (23.8) | 0.000 |
| Psychological (range 6-30) | 17.0 (16.0-19.0) | 24.0 (22.0-25.0) | 7.0 (41.2) | 0.000 |
| Social relationships (range 3-15) | 9.0 (8.0-10.0) | 12.0 (12.0-13.0) | 3.0 (33.3) | 0.000 |
| Environment (range 8-40) | 21.0 (18.0-24.0) | 29.0 (26.0-32.0) | 8.0 (38.1) | 0.000 |

^a^ Wilcoxon signed-rank test

**Telangana state**

|  | **Median and IQR baseline score (n=41)** | **Median and IQR follow-up sore (n=41)** | **Mean difference baseline vs follow-up (95%CI)** | **p-value^a^** |
| --- | --- | --- | --- | --- |
| Physical health (range 7-35) | 20.0 (18.5-21.5) | 21.0 (18.0-23.0) | 1.0 (5.0) | 0.601 |
| Psychological (range 6-30) | 16.0 (15.0-18.5) | 17.0 (15.0-19.0) | 1.0 (6.3) | 0.323 |
| Social relationships (range 3-15) | 7.0 (5.0-10.0) | 9.0 (7.0-11.0) | 2.0 (28.6) | 0.026 |
| Environment (range 8-40) | 19.0 (17.0-22.5) | 21.0 (19.0-24.0) | 2.0 (10.5) | 0.005 |

^a^ Wilcoxon signed-rank test
