# Supplementary material for: Strengthening individual and family resilience against leprosy-related discrimination: A pilot intervention study
Source: PLoS Negl Trop Dis. 2021 Apr 2;15(4):e0009329. doi: 10.1371/journal.pntd.0009329 (PMC8046345; doi:10.1371/journal.pntd.0009329)
Supplement: S3 Text — (DOCX) [file pntd.0009329.s004.docx]

**S3 Supporting information file****Factors associated with more short-term improvement**

Some factors were associated with more short-term improvements on the CD-RISC and WHOQOL-BREF scales. We developed two models for each state, given the large differences in mean improvement per state. We used bootstrapped stepwise multivariate linear regression with backward elimination (bootstrapping was performed to correct for non-normality of the data).

**Odisha state**
Multivariate analysis showed that participants with occupation ‘other’ had significantly less improvement on the CD-RISC between baseline and the first follow-up assessments (Table 1). This model explained 16% of the variability of increase in resilience score.

**Table 1. Correlations between absolute difference in CD-RISC scores between baseline and first follow-up in Odisha state, and the other variables in the dataset. This model explained 16% of the variability of increase in resilience score.**

|  | Regression coefficient | Standard error | p-value | 95% Confidence interval | |
| --- | --- | --- | --- | --- | --- |
| (Constant) | 32.733 | 1.583 | .001 | 29.801 | 35.908 |
| Occupation ‘other’ | -6.516 | 2.353 | .009 | -11.191 | -2.084 |

R-squared= 0.164

In addition, multivariate analysis showed that men had significantly more improvement on the WHOQOL-BREF between baseline and the first follow-up assessment (Table 2). This model explained 17% of the variability of increase in quality of life score.

**Table 2. Correlations between absolute difference in WHOQOL-BREF scores between baseline and first follow-up in Odisha state, and the other variables in the dataset. This model explained 17% of the variability of increase in quality of life score.**

|  | Regression coefficient | Standard error | p-value | 95% Confidence interval | |
| --- | --- | --- | --- | --- | --- |
| (Constant) | 28.294 | 1.561 | .001 | 25.250 | 31.266 |
| Female gender | -5.885 | 2.029 | .007 | -9.944 | -1.877 |

R-squared= 0.172

**Telangana state**Multivariate analysis showed that participants who were unemployed had significantly more improvement on the CD-RISC between baseline and the first follow-up assessments (Table 3). This model explained 24% of the variability of increase in resilience score.

**Table 3. Correlations between absolute difference in CD-RISC scores between baseline and first follow-up in Telangana state, and the other variables in the dataset. This model explained 24% of the variability of increase in resilience score.**

|  | Regression coefficient | Standard error | p-value | 95% Confidence interval | |
| --- | --- | --- | --- | --- | --- |
| (Constant) | -.298 | 1.384 | .832 | -3.120 | 2.379 |
| Occupation paid work | -4.535 | 1.938 | .030 | -8.338 | -.640 |
| Religion (Hindu) | 4.312 | 1.734 | .026 | .808 | 7.851 |

R-squared= 0.237

Multivariate analysis showed that persons affected and participants who were Hindu had significantly more improvement on the WHOQOL-BREF between baseline and the first follow-up assessments (Table 4). This model explained 50% of the variability of increase in quality of life score in Telangana state. Religion alone explained 40% of the variability (Table 5).

**Table 4. Correlations between absolute difference in WHOQOL-BREF scores between baseline and first follow-up in Telangana state, and the other variables in the dataset. This model explained 50% of the variability of increase in quality of life score.**

|  | Regression coefficient | Standard error | p-value | 95% Confidence interval | |
| --- | --- | --- | --- | --- | --- |
| (Constant) | -2.452 | 1.574 | .130 | -5.509 | .609 |
| Person affected | 6.300 | 2.563 | .023 | .917 | 11.041 |
| Religion (Hindu) | 11.112 | 2.071 | .001 | 7.057 | 15.268 |

R-squared= 0.495

**Table 5. Religion alone explained 40% of the variability of the absolute difference in WHOQOL-BREF scores between baseline and first follow-up in Telangana state.**

|  | Regression coefficient | Standard error | p-value | 95% Confidence interval | |
| --- | --- | --- | --- | --- | --- |
| (Constant) | -.952 | 1.697 | .579 | -4.222 | 2.318 |
| Religion (Hindu) | 11.502 | 2.205 | .001 | 7.019 | 15.717 |

R-squared= 0.401
