# Supplementary material for: Strengthening individual and family resilience against leprosy-related discrimination: A pilot intervention study
Source: PLoS Negl Trop Dis. 2021 Apr 2;15(4):e0009329. doi: 10.1371/journal.pntd.0009329 (PMC8046345; doi:10.1371/journal.pntd.0009329)
Supplement: Attachment — Submitted filename: Point-by-point reply_Resilience pilot v22-02-2021.docx [file pntd.0009329.s005.docx]

**Point-by-point reply document

Manuscript number**: PNTD-D-20-01904 **Manuscript title**: “Strengthening individual and family resilience against leprosy-related discrimination: a pilot intervention study”
**Deadline for submission of revision**: mid-April

| **Reviewer comment** | **Reaction authors** | **Changes in manuscript (highlighted in yellow)** |
| --- | --- | --- |
| Reviewer #2: Objective is quite clear and promising. Method (quasi-experimental) correctly selected for this purpose. The population has proper specificities that may restrict results to them or, of course, to social groups similar to them. However, the studied groups are clearly defined and described - therefore, no conflict arises from this particularity. | We thank the reviewer for this comment. | N/a |
| Reviewer #3: The objectives are clearly articulated. The authors have a clear direction as to what they intend to achieve as evident in their design and use of validated tools for assessing the outcomes. However, there are concerns as to why the authors decided to aggregate the scores of the individual domains of the WHO-QoLBREF tool as a measure of QoL. The scores from the QoL domains should be explored as intended by the tool.  and  Reviewer #3: The analysis plan is clear. However, it needs to be modified. The QoL tool used explores QoL in 4 dimensions as stated by the authors. It should not be modified without statistical reason; having been validated to explore QoL as intended. | We thank the reviewer for this comment. On line 197, we wrote “Even though there is no official total score for the WHOQOL-BREF [27], we calculated a sum score of 130 by adding the scores obtained on each of the 26 questions to be able to compare the total sum scores pre- and post-intervention.”. Unfortunately, the WHOQOL-BREF only has domain scores, and no total (sum) score is used. Because we wanted to measure the impact of the intervention on the construct of QoL (which consists of four domains), we decided to calculate a total score based on the four constructs of QoL.   We have also reported the domain scores in supporting information file 2. Kindly see line 290-293 (“an overview of the difference pre- and post-intervention per question of the CD-RISC and WHOQOL-BREF and of the difference in domain scores on the WHOQOL-BREF can be found as supporting information file.”) and line 300-303 (“In Odisha state, all domains of the WHOQOL-BREF (physical health, psychological, social relationships and environment) significantly improved post-intervention. In Telangana state, only the domains social relationships and environment significantly improved post-intervention (supporting information file 2).”). | To clarify this point, supporting information file 2 has been updated, the median scores instead of the means have been used to calculate the domain scores on the WHOQOL-BREF. In addition, instead of looking at the mean improvements per question for the CD-RISC and WHOQOL-BREF, we have looked at the median improvement per question. We have presented this graphically instead of in a table. |
| Reviewer #3: The authors state that the covid-19 pandemic was a limitation to the study. It is not exactly clear how this happened and the dimension of the effect of the work. The authors seem to say there was an effect on the accuracy of the findings because of this. This should either be revised or clearly shown how. | We thank the reviewer for this comment. We have discussed the COVID-19 pandemic on line 460-463: “Importantly, the intervention also coincided with the COVID-19 pandemic and restrictions in Telangana (which were not as severe in Odisha). It is possible that the psychosocial nature of the intervention was not substantial enough to address their more fundamental and overwhelming needs.” Unfortunately, it is very difficult to determine the specific impact of the pandemic beyond asserting that there was delay in planned data gathering where there was lockdown, Teleganga, the urban sample whereas the rural area was less disturbed. Unfortunately, the exact nature of the effect of the pandemic on our findings remains unclear, but should be acknowledged |  |
| Reviewer #1: Participants and sampling procedure. From line 153 to 159 authors should provide further important information such as disability degree of treated leprosy cases and time after treatment and type of treatment (multibacillary or paucibacillary accordint to WHO), because those who have disabilities and some skin alterations will suffer stigma as compared with those 0 degree and that did not treat with Multibacillary schemes. Authors should make clear that they used the participant as his/her own control along the different times of intervention. *And* Reviewer #1: What is the data regarding disability grade? It is very important to discuss about resilience. Authors should provide data about type of treatment, whether multibacillary or paucibacillary. Clofazimine can cause ichthyosis and change in skin colour what will interfere in stigma and resilience of these individuals. | We thank the reviewer for this comment and fully agree that visible impairment (disability grade) often results in greater stigma. We have reported the disability grade on line 262-265: “(...) Most persons affected (n=16, 70%) had grade 2 disabilities, followed by grade 0 (n=5, 22%) and grade 1 (n=4, 17%). Most persons affected were diagnosed between five and ten years ago (n=14, 61%). Only one participant (n=1, 4%) was diagnosed over ten years ago (…)”. Since our participants had already completed treatment, there was nothing to report on treatment. We do agree with reviewer 1 that this would be an important topic to discuss should participants still be on treatment. | - |
| Reviewer #1: From line 167 to 172 I agree with authors, however, for the subsequente study, the sample size should be calculated using a the effect size found in this pilot study through a statistics software.   Ref: Moore CG, Carter RE, Nietert PJ, Stewart PW. Recommendations for planning pilot studies in clinical and translational research. Clinical and translational science. 2011;4(5):332-7. | We thank the reviewer for his/her comment. Since this was an exploratory pilot study, we considered a calculation based on effect sizes not appropriate. We therefore decided not to make any changes to our sample size calculation, we have followed the guidelines of Hertzog for the calculation of the sample size. | - |
| Reviewer #1: Data collection From line 175 to 180 authors should rewrite and changing some words, for instance, gender and education, since that sex and schooling are more appropriated.  “Sex refers to the biological distinctions between males and females, most often in connection with reproductive functions. Gender, by contrast, emphasises the socially constructed differences between men and women that give rise to masculinity and femininity.” Ref: Short SE, Yang YC, Jenkins TM. Sex, gender, genetics, and health. American journal of public health. 2013;103 Suppl 1:S93-101. Regarding schooling, it refers to a formal process related to institution of education. I think authors are using this meaning for this word . | We agree with the participant that in this case “sex” is more appropriate than “gender”, as we have asked participants about their sex. In this context we prefer “education” over “schooling” since it includes post-school education such as technical education, noted as “higher” in our data collection.– We have specified the type of education in Table 1 (no or no formal, primary, secondary or higher). | “Gender” has been replaced by “sex” in Table 1, the results and the methods section. |
| Reviewer #1: From line 182 to 206 I recommend to authors to create a new subtitle called “Research questionnaires” or similar. From line 208 to 2014 I suggest to authors create a subtitle named “Phases of follow-up time” or similar for describing these different times of follow-up. | This has been revised. | On line 175, a level 3 heading has been added “data collection tools”. On line 209 we have added a level 3 heading “phases of follow-up”. |
| Reviewer #1: Data analysis From line 219 to 231, authors wrote about the data analyses. I recommend them starting this section statint in relation to normality tests applied, since that questionaires which provide ordinary scores the figures have as a result non-parametric distribution. Thus, authors may use Wilcoxon for two medians or Friedman test for more than 2 medians when it deal with paired method. Afterward, authors should quote the statistical tests utilised to found out these findings. I suggest the use of Binomial test for comaparing the difference between proportions showed in table 1. | We thank the reviewer for these helpful observations and suggestions. We apologise for the mistakes we made in our data analysis and have corrected this accordingly. | Methods > “Data collection” has also been revised:  “Quantitative data were collected on paper forms and entered in a database created using Epi Info. The data were analysed using IBM SPSS Statistics 24. Differences between participants from Odisha and Telangana state were evaluated using the Mann-Whitney U test for continuous variables, X2 statistics for categorical variables and Fisher’s Exact test for categorical variables for which the expected values in one of the cells of the contingency table was less than five. Frequencies and percentages were calculated to depict demographic information of the participants. Median total scores and the interquartile range (IQR) of the CD-RISC and WHOQOL-BREF were calculated for the different participant groups to summarize resilience and quality of life scores pre- and post-intervention. The Wilcoxon signed-rank test was performed to check whether the scores were significantly different pre- and post- intervention. (…)” |
| Reviewer #1: Authors should quote the manufacturer and version of statistical tests used in this analysis in a complete sentence (not only SPSS). | This has been added. | Line 220: “The data were analysed using IBM SPSS Statistics 24” |
| Reviewer #1: Ethical considerations Is there a protocol number or approving nunber for this research provided by Institutional Ethics Committee of the Lepra-Blue Peter Public Health and Research Centre? | The approval number for the study is IEC/LEPRA/2019/05. | - |
| Reviewer #1: Results In accordance with data showed in table 1. Authors should use the binomial test for comparing differences between Odisha and Telangana states proportions. Whether authors are interested in prove the diferences between these two groups they have starting showing diferences among demografic informations.  Are the the sex, religion, occupation associated with any group? The binomial test or another association test will respond this question. For each comparison authors should provide p-value. | We thank the reviewer for this suggestion. This has now been added.   For age (continuous data, non-normally distributed) we have used the Mann-Whitney U test. For gender, occupation, education and participant type (categorical variables) we have used the X2 statistic. For living area, language and religion (categorical variables, some with observed values less than 5) we have used Fisher’s Exact test. | A column has been added to Table 1. The footnote includes the tests used.  Methods > “Data collection” has also been revised:  “Quantitative data were collected on paper forms and entered in a database created using Epi Info. The data were analysed using IBM SPSS Statistics 24. Differences between participants from Odisha and Telangana state were evaluated using the Mann-Whitney U test for continuous variables, X2 statistics for categorical variables and Fisher’s Exact test for categorical variables for which the expected values in one of the cells of the contingency table was less than five. Frequencies and percentages were calculated to depict demographic information of the participants. Median total scores and the interquartile range (IQR) of the CD-RISC and WHOQOL-BREF were calculated for the different participant groups to summarize resilience and quality of life scores pre- and post-intervention. The Wilcoxon signed-rank test was performed to check whether the scores were significantly different pre- and post- intervention. (…)” |
| Reviewer #1: The standard deviation suggest that age data are non-parametric. Hence, the authors shall never use mean, however they should use median to represent the age. | We apologise for this mistake and thank the reviewer for this observation and comment. Age is indeed non-normally distributed. We have therefore calculated the median and IQR (instead of mean and SD). | Please see Table 1. |
| Reviewer #1: According to table 2 and  3, I have noticed they are utilising the same data. Nevertheless, the table 3 compared three averages. Therefore, authors should make one table summarising the table 2 and 3. It is more accurate compare the 3 averages instead 2 averages (or difference of mean rank).  Whether authors test the normality and consequently the data will be non-parametric, what I believe, they should use the Friedman test for comparing 3 or more follow-up times considering the participant as his/her own control. If the distribuition passed in normality test, mean and SD should be provided. Otherwise, authors must use median, maximum and minimum values found for each median. On the other hand, I have noted that authors made a mistake using t-test not for the reason associated with normality test, however for the fact they compare 3 different follow-up times by means of t-test, since that it may represent a type I error. The comparison encompassing, 3 or more groups o follow-up times, paired, must be lead by use o Friedman or ANOVA paired if the distribuition is normal. I recommend that authors should remake the data analyses, what will obligate them rewrite some parts of results and discussion. | We thank the reviewer for this comment. We have revised Table 3 and have updated the information with medians instead of mean scores. We have used the Wilcoxon signed-rank test to calculate the differences between baseline and follow-up 1 (same as Table 2) and baseline and follow-up 2. We did not use Friedman’s test, because we did not want to show a trend, but rather wanted to calculate whether the effect of the intervention was maintained at second follow-up. Therefore, we have also omitted the column that showed the p-value of the first versus second follow-up, since this is not meaningful information. Thank you.  Table 2 has also been revised. We have calculated the median instead of the mean scores. The p-value has been calculated using the Wilcoxon signed-rank test. | Table 2 and 3 have been revised. In addition, the figures have been updated in the text using track changes.  Methods > “Data collection” has also been revised:  “Quantitative data were collected on paper forms and entered in a database created using Epi Info. The data were analysed using IBM SPSS Statistics 24. Differences between participants from Odisha and Telangana state were evaluated using the Mann-Whitney U test for continuous variables, X2 statistics for categorical variables and Fisher’s Exact test for categorical variables for which the expected values in one of the cells of the contingency table was less than five. Frequencies and percentages were calculated to depict demographic information of the participants. Median total scores and the interquartile range (IQR) of the CD-RISC and WHOQOL-BREF were calculated for the different participant groups to summarize resilience and quality of life scores pre- and post-intervention. The Wilcoxon signed-rank test was performed to check whether the scores were significantly different pre- and post- intervention. (…)” |
| Reviewer #1: From line 225 to 229 it was not clear what type of regression model was used in this analysis. Linear reagression accept only normal distribuition (it requeres normality test application). Did the authors use the Logistic Regression? The text must be written clearly. and Reviewer #1: From line 296 to 315, authors showed results about multivariate analysis by means of Regression model such as seen in supporting file 3. As these findings deal with scores given throught a Questionnaire, I enphasise that normality data should be provided and generally one measure, represented by a score, is not a parameter for another, so it should be considered as a non-parametric variable. The analysis in supporting file 3 did not show where authors find the R square for these data. If the model  called by authors another occupation than paid work and participants who were Hindu can explain 24% of variability of increase in resilience for CD-RISC, the R square or correlation coefficient should be given by authors in supplementary data. | We thank the reviewer for this important comment. We apologise for not having clarified what kind of multivariate regression we performed, we performed (bootstrapped) multivariate linear regression. We have changed this accordingly. We performed bootstrapping to correct for non-normality. We have now clarified that in our paper and S3. We have checked with a statistician whether this was the appropriate analysis for our data, he confirmed.  In addition, while r-squared was mentioned in S3 in the Table heading, this may not have been very clear. We have therefore now added r-squared below each Table for clarity. Finally, we have added the model that shows the influence of religion on quality of life in Telangana state (Table 5 in supporting information file 3). | Supporting information file S3 was updated.   The following has been changed in methods > data analysis (line 230-235): “In addition, we created a new variable that contained the absolute difference between the baseline and follow-up scores of the CD-RISC and WHOQOL-BREF and used bootstrapped stepwise multivariate linear regression with backward elimination to see if there were associations between these variables and the other variables in our dataset (~~gender~~sex, age, participant type, occupation, education, role in the family, and in Telangana state religion also). Bootstrapping was performed to correct for non-normality of the data. (…)” |
| Reviewer #2: A most welcome study on stigma, discrimination and resilience in leprosy. The selection of urban and rural area with a reasonable low detection rate was also quite opportune to reproduce a social environment  that may resemble the average behavioral condition of  averge villages in leprosy endemic countries anywhere. Studying areas with very high or close to zero detection rate would give non reproducible results to be widely applied. In addition, on should consider that, for a study on stigma/discrimination issues, it is interesting to note that 70% had DG2, that is, visible deformities, that is, they were prone to easily be recognized as leprosy patients. This grants value to the results of the study. | Thank you. | N/a |
| Reviewer #2: Also opportune was the inclusion of human rights issues in the action learning process, since to me it seems crucial as part of addressing resilience aspects of stigmatized people. | Thank you. | N/a |
| Reviewer #2: For practical purposes, the association of the bamboo and its vegetal characterizes to resilience concept was a marvelous decision. Indeed, participants acknowledged this. | Thank you. | N/a |
| Reviewer #2: Though one understand the basic and/or advanced differences between Muslim and Hindu creed, it is estimated that profession of these creeds in a same ethnological culture has some interweaving’s. This leads to the fact that religion is a relevant variable to be discussed in this study, what was done scarcely – unfortunately. As a matter of fact, from the article we learn that in Telangana state both CD-RISK and WHOQOL had significantly more improvement among Hindus between baseline and the first follow-up assessments. This finding required a more detailed discussion!  In a future study, religion as a variable should be throughout explored. | We thank the reviewer for this comment. We have added a few lines to our manuscript. | The following was added to line 509-514: “As noted above, since this pilot study included relatively small numbers of participants across various religious groups, and coincided with religious persecution due to the ‘Citizenship Amendment Act’, interpretation of these data must be cautious. However, in response to these indications and current literature on this topic [11], there is great need for more detailed exploration of the interplay between resilience and religion, beliefs, spirituality and faith practices.” |
| Reviewer #2: The presentation of Results and Discussion may require some revision - please, a text length reduction applies. There are, to my personal feeling, excessive description of the findings of referred articles. This can be helpful to those not willingly to go for the entire cited article, but may render the reading of the original article tiresome. One example, but not the only: line 419. In Odisha, this (line 420) improvement was maintained at six-month follow-up. Singh and colleagues [51], who used the CD ....What follows is an almost full description of the referred article including figures! There are other citations such as this along the Discussion that, to my view, should be restricted to the direct liaison with the author's sentence and limited to the reference. Details of the referred article should not necessarily by reproduced within the article. Or, at least, make a selection of those references you find truly necessary. | This has been revised. | The following has been changed, line 432-439: “~~Singh and colleagues [51], who used the CD-RISC to assess resilience among elderly patients attending the geriatric outpatient clinic in Maharashtra state in India, found scores of 47.7 (elderly patients with depression), 55.5 (elderly patients with rheumatoid arthritis) and 77.4 (control group, elderly people who accompanied patients).~~ The baseline score of the participants in Odisha and Telangana state in the present study, ~~46.7 and 49.3,~~ were similar to that of elderly patients with depression in a study in Maharashtra state in India [51]. Follow-up scores in Odisha state ~~(75.5 and 72.0)~~ were similar to that of healthy, elderly controls in the same study [51], but somewhat higher than the scores found in two cross-sectional studies in Karnataka state among parents of children with intellectual disability and adult offspring of parents with schizophrenia [52,53].” |
| Reviewer #3: Study addresses and articulates what it intends to solve. | Thank you. | N/a |
